# *Acanthaster planci* Outbreak: Decline in Coral Health, Coral Size Structure Modification and Consequences for Obligate Decapod Assemblages

**DOI:** 10.1371/journal.pone.0035456

**Published:** 2012-04-17

**Authors:** Matthieu Leray, Maxime Béraud, Arthur Anker, Yannick Chancerelle, Suzanne C. Mills

**Affiliations:** 1 Laboratoire d'Excellence “CORAIL”, USR 3278 CRIOBE CNRS-EPHE, CBETM de l'Université de Perpignan, Perpignan, France; 2 Laboratório de Ciências do Mar – Labomar, Universidade Federal do Ceará, Fortaleza, Ceará, Brasil; 3 Laboratoire d'Excellence “CORAIL”, USR 3278 CRIOBE CNRS-EPHE, CRIOBE, Moorea, Polynésie française; University of Otago, New Zealand

## Abstract

Although benthic motile invertebrate communities encompass the vast majority of coral reef diversity, their response to habitat modification has been poorly studied. A variety of benthic species, particularly decapods, provide benefits to their coral host enabling them to cope with environmental stressors, and as a result benefit the overall diversity of coral-associated species. However, little is known about how invertebrate assemblages associated with corals will be affected by global perturbations, (either directly or indirectly via their coral host) or their consequences for ecosystem resilience. Analysis of a ten year dataset reveals that the greatest perturbation at Moorea over this time was an outbreak of the corallivorous sea star *Acanthaster planci* from 2006 to 2009 impacting habitat health, availability and size structure of *Pocillopora* spp. populations and highlights a positive relationship between coral head size and survival. We then present the results of a mensurative study in 2009 conducted at the end of the perturbation (*A. planci* outbreak) describing how coral-decapod communities change with percent coral mortality for a selected coral species, *Pocillopora eydouxi*. The loss of coral tissue as a consequence of *A. planci* consumption led to an increase in rarefied total species diversity, but caused drastic modifications in community composition driven by a shift from coral obligate to non-obligate decapod species. Our study highlights that larger corals left with live tissue in 2009, formed a restricted habitat where coral obligate decapods, including mutualists, could subsist. We conclude that the size structure of *Pocillopora* populations at the time of an *A. planci* outbreak may greatly condition the magnitude of coral mortality as well as the persistence of local populations of obligate decapods.

## Introduction

A combination of natural and anthropogenic disturbances is responsible for habitat modification, loss, and fragmentation. Habitat perturbation is a major concern for conservation and management, due to the concomitant decrease in biodiversity and abundance often accompanied by shifts in community structure which affects ecosystem functioning [1], [2], [3]. The response of ecological communities is determined by both the modification in habitat characteristics (e.g. availability, structural complexity) and the resource specificity of organisms within the community [4]. Specifically, a species response to habitat perturbation will vary according to their level of ecological specialization [5]. Generalists are typically less sensitive to habitat modification, because they are able to freely move throughout the landscape to colonize a wide range of territories, whereas habitat specialists are often highly dependent upon the distribution and availability of their habitat, making them more susceptible to habitat modifications [6], [7].

The importance of understanding how perturbations affect biogenic habitats that are dominant primary producers and serve as essential resources for whole communities (i.e. foundation species such as kelp, trees, or corals) is particularly critical because these habitats underpin the entire ecosystem. Most inhabitants of tropical coral reefs (fishes and invertebrates) are particularly vulnerable as they are directly or indirectly dependent on stony corals, which are in decline worldwide [8], [9]. Previous studies suggest that loss of coral cover can severely affect the diversity, abundance and composition of reef fish communities. For example, in Papua New Guinea an 8 year decline in coral cover from 70% to <25% was accompanied by a decline in over 75% of the observed fish species richness [10] with significant reductions in the settlement and recruitment of coral associated fish species [11]. In the meantime, declines in coral cover led to algal colonization and an increase in herbivore biomass and diversity [12]. Generally, habitat loss modifies the composition and trophic structure of fish assemblages with an overall loss of diversity [10].

Despite the vast majority of reef diversity being comprised of motile benthic invertebrate communities, their basic ecology, including their response to habitat modifications, has been poorly studied relative to reef fishes [13]. One explanation may be that most invertebrate species are cryptic and difficult to identify [14]. A variety of invertebrates live in corals of the genus *Pocillopora* (Pocilloporidae), which are important reef-builders in the Indo-Pacific region and typically provide food (including coral mucus) and structural habitat for multiple inhabitants, mostly decapods [15], [16], [17]. Some decapods are known to be coral obligate exosymbionts [18] providing essential benefits to their host, including the ability to cope with environmental stressors [19]. For example, some coral crabs (Trapeziidae) and snapping shrimps (Alpheidae) can increase the survival and growth of their host by actively defending the coral against corallivorous seastars [20], [21], [22], clearing sediments [23], [24] and ameliorating negative effects of vermetid snail nets [25]. These symbionts have been considered “habitat-maintaining” species, because they enable the persistence of *Pocillopora*, which are “habitat-forming” species [26]. The positive direct effects of decapods on coral growth and survival may provide positive indirect benefits to other coral-associated species [26] and thus decapods may contribute to the resistance of coral reefs faced with natural and anthropogenic stress. Despite their potential importance for foundation species and thus the ecosystem, only a limited number of studies have looked at the effect of perturbations on coral decapods [27]–[30].

We aimed to identify an important perturbation affecting corals at our study site, the island of Moorea, French Polynesia, and then determine the consequences of this perturbation on coral decapod communities. Coral reefs at Moorea have experienced recurrent perturbations since 1981, including six bleaching events in 1984, 1987, 1991, 1994, 2002 and 2003 and two outbreaks of *Acanthaster planci* between 1980–1982 and 2006–2009 [31]. Coral assemblage was considered resilient after the last four bleaching events in terms of coral cover [31]. However, the effects of the more recent *A. planci* outbreak on coral assemblage and their associated decapods is not known.

Long-term declines in coral cover throughout the Indo-Pacific are commonly caused by frequent outbreaks of the corallivorous crown-of-thorns sea star, *A. planci* [32]. *Acanthaster planci* outbreaks vary greatly in their effect on coral communities [33] with up to 90% loss in coral cover reported on the Great Barrier Reef [34], Guam [35], and Southern Japan [36], compared to a negligible impact, so far, on coral communities in Hawaii [37]. *Acanthaster planci* typically attacks a coral head from the top, extruding its stomach and releasing soft tissue-digesting enzymes, leaving a distinctive scar. Coral loss during an outbreak is primarily influenced by both coral density (as *A. planci* show feeding preferences for certain species of coral) [38]–[40] and the presence and identity of coral symbionts that defend their hosts from attack [20]–[22], [41]. Causes of population outbreaks remain largely unknown and are likely the result of a combination of several factors [42] including terrestrial run-off, overfishing of predators and increased sea-surface temperatures, which are all thought to promote the survival of pre- and post-settlement sea stars [43]–[45]. The end of an outbreak occurs when most palatable prey are consumed [46], rather than a density-dependent epidemic [47].

In this paper we aim to document patterns of *Pocillopora* mortality by analyzing a ten year sequence of permanent quadrats from 2000 to 2009 encompassing two bleaching events in 2002 and 2003 and an *A. planci* outbreak from 2006–2009. Secondly we describe how coral-decapod communities change across a natural gradient of mortality for a selected coral species, *Pocillopora eydouxi*, at the end of the *A. planci* outbreak in 2009. We describe the consequences of habitat modification on the distribution of a cryptic yet highly diverse and functionally important fraction of coral reef biodiversity. Finally, we discuss the functional importance of shifts in decapod communities for the recovery of local coral reef communities following an outbreak of *A. planci*.

## Materials and Methods

### Ten year survey of *Pocillopora* populations

The study was conducted on the outer reef slope of Moorea, Society Archipelago, French Polynesia (17°30′S, 149°50′W), using 60 permanent 1 m^2^ quadrats placed at three sites (20 quadrats/site between 10 and 15 m), Vaipahu and Tiahura on the north coast and Haapiti on the west coast (see [48] for site description and sampling methodology). High resolution digital photos were taken from the permanent quadrats every two years between 2000 and 2009 and analyzed. Most coral heads of the genus *Pocillopora* could not be reliably identified to the species level from the photographs, but, *P. eydouxi*, *P. woodjonesi*, *P. verrucosa*, *P. elegans* and *P. meandrina* are known to occur among the corals at our survey site.

We quantified three parameters: 1) changes in habitat “health” (live vs. dead *Pocillopora*), 2) changes in habitat availability (density of *Pocillopora*), and 3) changes in habitat size structure (size of *Pocillopora*). It is important to note that in 1) and 3) we consider *Pocillopora* as a habitat in terms of its branching structure, independently of the presence/absence of living tissue. For each quadrat, we first counted the number of *Pocillopora* (density) that were then classified into three categories: “live” (>98% live coral), “partially dead” (98% to 2% live coral) and “completely dead” (<2% live coral). In practice, corals were classified as live or dead corals when we could not detect any dead or live tissue respectively. Nevertheless, we assume that minor proportions of live or dead tissue (<2%) might not have been noticeable from photographs. Finally, we measured the total surface area (2D aerial surface in cm^2^) of all *Pocillopora* (size) using VidAna 1.0. [49] (see www.marinespatialecologylab.org/resources/vidana/ for further details).

### Decapod communities across a natural gradient of percent coral mortality

The study was conducted on the outer reef slope of Moorea at a depth range of 5–8 m along a 100 m stretch extending west from Opunohu's Pass along the northern shore of Moorea [50]. We surveyed decapods living on *Pocillopora eydouxi* because it was the most common coral species on the outer reef slope of Moorea at the time of collection. In order to describe the indirect effects of the *A. planci* outbreak (due to consumption of coral tissue) on decapod communities associated with *P. eydouxi*, decapods were counted on 52 non-eaten corals (100% live), 22 partially eaten corals (showing *A. planci* feeding scars and partial tissue consumption), and 8 completely dead corals. Sampling was conducted between May and July 2009, a period at the end of the outbreak of *A. planci*. Importantly, decapods inhabiting *Pocillopora* in Moorea show no seasonal variation in community structure [15].

Intraspecific variation in coral head structure has been linked to physical factors such as water movements [51] which can affect associated communities [52]. Therefore, because we were primarily interested in isolating the effect of coral tissue loss on associated coral decapod communities, we controlled for coral host shape. We used a measuring tape to estimate the maximum diameter (L), perpendicular diameter (l) maximum height (h) and interbranch space of each coral head prior to collection in order to meet certain morphological criteria: branch length (15–20 cm), interbranch space (3–5 cm), and shape (dome-shaped). We estimated the volume of live/dead tissue of coral heads using the formula for the volume of an ellipsoid: 4/3 π×L×l×h, which has previously been shown to be a good proxy for “living space” of *Pocillopora* [15], [53].

Decapods living on non-eaten corals and partially eaten/completely dead corals were sampled using different methods, non-destructive and destructive respectively. The non-destructive method entailed a single immersion of each of the 52 non-eaten corals in a low concentration clove oil solution (0.02%), which enabled an exhaustive sampling of decapods while minimizing coral death (see Appendix S1 for description and validation of sampling methodology). However, because brachyuran crabs hold onto algae or retreat into deep crevices in the dead part of corals, partially eaten and completely dead corals could not be efficiently sampled using the non-destructive clove oil method alone. Therefore, these corals were broken down into smaller pieces and all decapods were extracted by hand. In order to limit the destruction of coral reef habitat as a result of this sampling technique, we did not sample as many partially eaten (22) or completely dead (8) corals compared to non-eaten corals (52). Approval was granted from our institutional animal ethics committee (CNRS - Permit Number: 006725).

All decapod specimens were identified under a dissecting scope to the lowest taxonomic level possible, in most cases to genus or genus/species level, but in some cases only to family level. Whenever possible, morphospecies were recognized and taken into account. Decapods at early juvenile stages were not considered as they could not be confidently identified by visual inspection alone (<5% total number of individuals). Specimen abundance – total number of specimens of a species per colony – was scored.

### Ecological classification of decapod species

Communities of decapods that use corals as a unique habitat (coral specialized) are well characterized in the Pacific and Moorea [15], [53]–[56]. Some species occurring in Moorea are known to provide some benefits to their host; this includes species of coral crabs, *Trapezia serenei*, *T. guttata*, *T. septata*, *T. flavopunctata*, and the snapping shrimp *Alpheus lottini* [56]. In some cases, species are known to be coral specialized but the biological interactions between the associate and coral host remain completely unknown; this is the case for the pontoniine shrimps *Fennera chacei* and *Harpiliopsis* spp. found exclusively in association with living *Pocillopora* spp. [57]. Therefore, because the true nature of the association with corals has only been established for a few species (i.e. only a few species of the genus *Trapezia* are known to be coral mutualists), we chose to refer to species which have been documented to live only on live corals (specialists) as “coral obligate species” (rather than symbionts or mutualists) and to species using a wider range of habitats (generalists or opportunists) as “non-obligate species” [53]. Coral obligate species occupy and feed on live coral tissue or mucus and organic particles trapped in them, but usually do not use dead parts of the coral head colonized by either algae, sponges or other encrusting organisms [54]. On the other hand, non-obligate species may occupy any habitat *per se* including live coral tissue [53].

### Statistical analyses

Our sampling strategy was skewed with a higher number of non-eaten coral (52) compared to partially eaten (22) and completely dead corals (8). In order to evaluate our sampling effort among non-eaten, partially dead and completely dead corals, expected species accumulation curves with 95% confidence intervals (1000 randomizations sampled with replacement) were computed using the program EstimateS [58], [59].

We investigated the effect of tissue consumption by *A. planci* on the diversity of decapods living on *Pocillopora* using descriptive statistics. First, we drew the central tendency (mean fit) of the relationship between proportion of coral tissue, total richness and total rarefied richness using linear regressions. Species richness is known to increase with the number of individuals and substantial variation existed in the abundance of decapods in corals among our sampling groups. Therefore to account for differences in decapod abundance between corals, we used individual rarefaction [60] (rarefying each sampled coral to the minimum abundance observed using R 2.12.2 [61], package ‘Vegan’ [62]). The significance of regression models was tested using a one-way ANOVA and the Akaike's information criterion was used to determine the linear model best fitting the data. A similar least square regression approach was used to describe the relationship between coral obligate and non-obligate species richness and the decrease in coral tissue.

We also examined variation in species composition between non-eaten, partially dead and completely dead *Pocillopora* using beta diversity metrics. Because differences in beta diversity can be driven by changes in species incidence (presence-absence) or relative abundance, we used: 1) Jaccard, an incidence based metric, and 2) Bray-Curtis, an abundance based metric. Both indexes are bound from 0 to 1, where 0 means that communities have identical composition and 1 means that communities do not share any species. Dissimilarity matrices were calculated among all pairs of *Pocillopora* and non-metric multidimensional scaling (NMDS) was used to examine patterns of ordination of objects (*Pocillopora*) from different groups (non-eaten, partially eaten and completely dead *Pocillopora*) in 2 dimension plots. Objects that are different in species composition are plotted far apart, whereas similar objects are placed close together. Nonparametric multivariate permutation tests were computed: 1) PERMANOVA [63] tests for differences in the position of sets of objects in multivariate space, 2) PERMADISP [64] tests for differences in beta diversity values between groups of objects (differences in variation around the multivariate mean).

## Results

### Ten year survey of the *Pocillopora* populations

#### Change in habitat health

**Figure 1:**
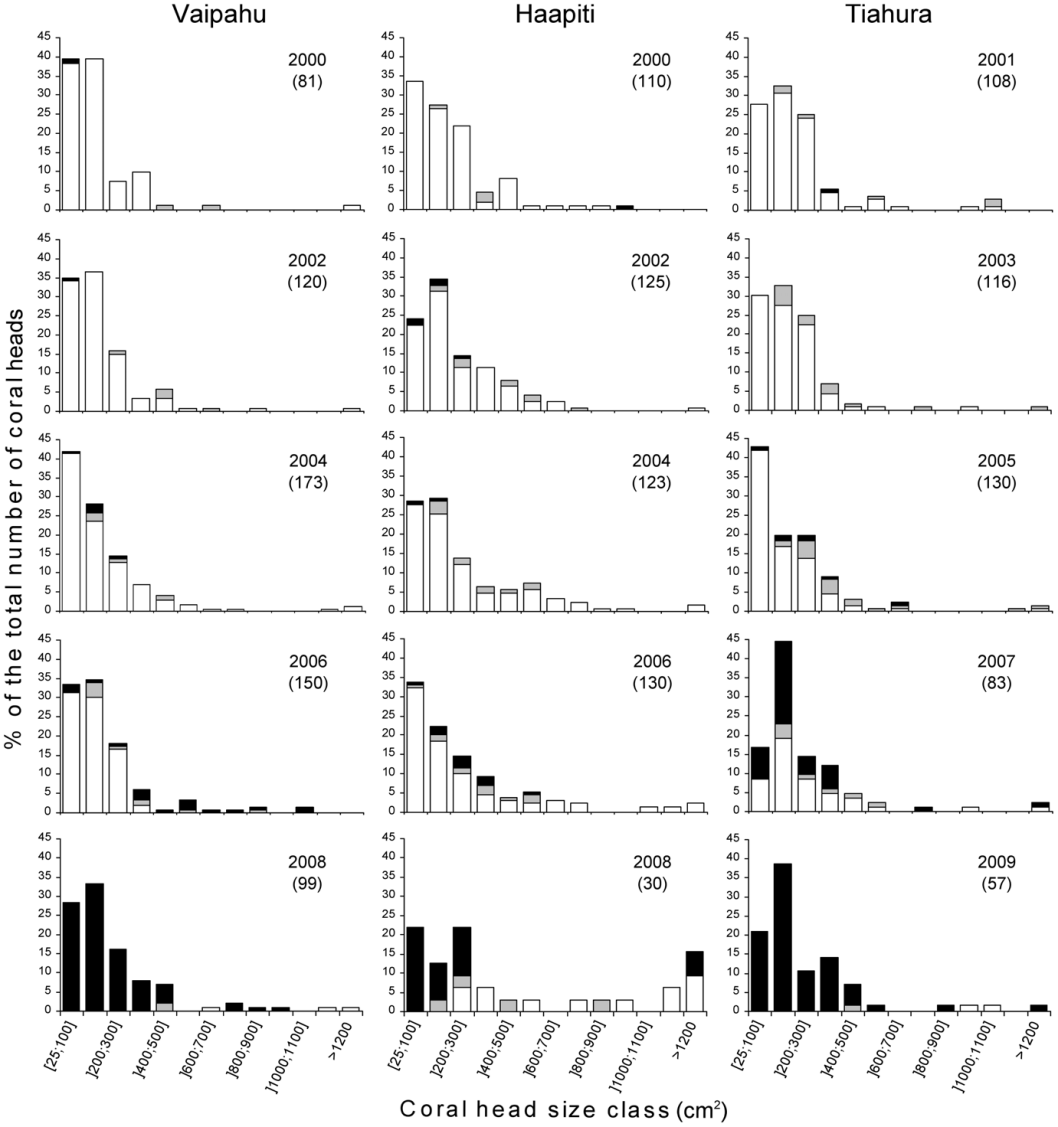
Effects of a recent outbreak of *Acanthaster planci* from 2006–2009 on *Pocillopora* populations. Size-class (cm^2^) distributions of live (white), partially dead (grey) and completely dead (black) corals (*Pocillopora* spp.) as a proportion of the total number of coral heads at each site. High resolution photographs of 60 permanent quadrats were taken at three sites in Moorea (20 quadrats/site) every two years from 2000 to 2008 at the sites Vaipahu and Haapiti and from 2001–2009 at Tiahura. Numbers in parentheses represent the total number of *Pocillopora* measured per site.

Live *Pocillopora* were highly dominant at the three study sites from 2000 to 2005 (80–96%; Fig. 1). Only minor mortality was observed in 2004 at Vaipahu and Haapiti (3% and 2% respectively) and in 2005 at Taihura (5%) prior to and after the 2002 and 2003 bleaching events. However, significant effects of the 2006–2009 *A. planci* outbreak were already detected in 2007 at Tiahura with a 37% increase in the proportion of dead *Pocillopora* and the presence of distinctive feeding scars from *A. planci* on partially dead corals. Large decreases in the proportion of live coral were detected in 2008 and 2009 at all three sites (a decrease of 77%, 48% and 77% at Vaipahu, Haapiti and Tiahura respectively). Completely dead corals became highly dominant at the three study sites in 2008–2009, with the presence of only a few larger corals that had not been eaten. All partially dead *Pocillopora* in 2008–2009 had been partially consumed by *A. planci*, as they all showed feeding scars and mortality only at their branch tips, whereas internal parts of the colony, which cannot be reached by the extruded *A. planci* stomach, remained alive.

#### Change in habitat availability

There was no decrease in the total number of *Pocillopora* before and after the two bleaching events from 2002–2003 to 2004–2005 (120 to 174, 125 to 123 and 116 to 131 at Vaipahu, Haapiti and Tiahura, respectively). On the other hand, there was an overall loss of habitat availability after the *A. planci* outbreak at the three sites (150 to 99, 130 to 30 and 130 to 57 *Pocillopora* at Vaipahu, Haapiti and Tiahura, respectively).

#### Change in habitat size structure

**Figure 2:**
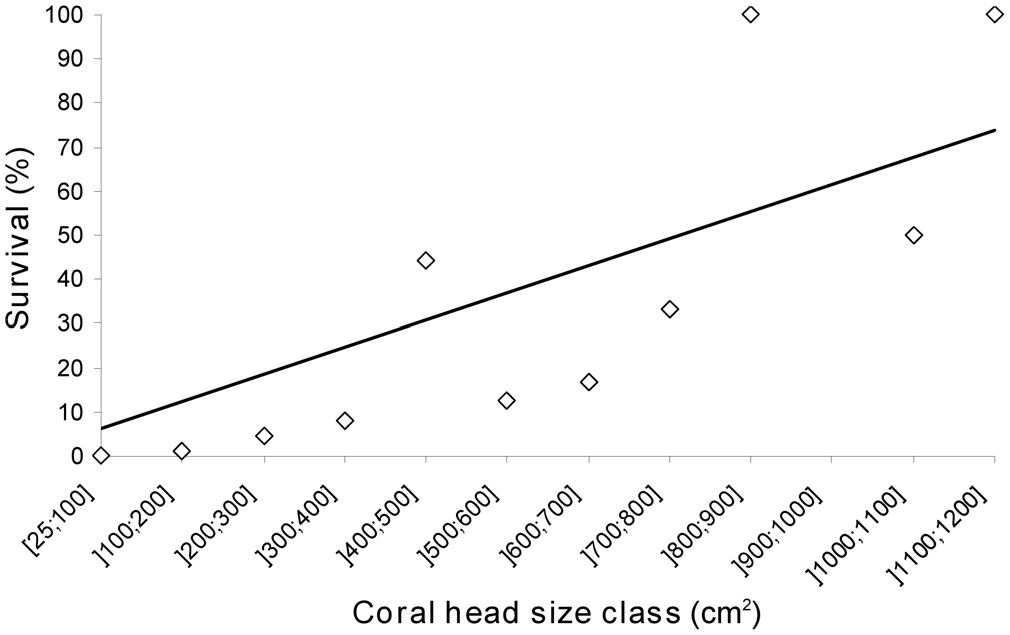
Proportion of coral that survived the 2006–2009 outbreak of *A. planci* for each coral size class. Survival for each size class was calculated from (the number of live and partially dead *Pocillopora* in 2008–2009)/(the number of live and partially dead *Pocillopora* in 2005–2006)×100. Counts of *Pocillopora* at the three study sites were combined. Linear regression: *y* = 6.14*x*; *R^2^* = 0.61, N = 11, *p* = 0.002.

There was a high prevalence of small corals at the three study sites from 2000 to 2005 (Fig. 1). The two smallest size classes measuring 25 to <200 cm^2^ represented 61–77% and 51–60% of all *Pocillopora* spp. between 2000 and 2006 at Vaipahu and Haapiti respectively and 51–60% between 2001 and 2005 at Tiahura. However, the survival of *Pocillopora* during the outbreak of *A. planci* was positively and linearly related to coral size (Fig. 2). At the end of the outbreak in 2008–2009, corals measuring less than 200 cm^2^ were all completely dead or partially dead. The seven largest size classes measuring 600 to >1200 cm^2^ represented 3%, 10% and 2% of all live *Pocillopora* spp. in 2004–2005 at Vaipahu, Haapiti and Tiahura respectively. However, at the end of the outbreak in 2008–2009, these largest size classes represented 100%, 58% and 100% of all *Pocillopora* respectively.

### Response of decapod assemblages to consumption of their coral host tissue

There was little variation in size between sampled coral heads (mean ± SE = 8019±195 cm^−3^) and coral size was not significantly different between non-eaten, partially eaten and completely dead corals (One way ANOVA: *F*_2, 79_ = 0.31; *p* = 0.73). The number of corals sampled was sufficient to describe the community of coral obligates both in non-eaten and partially dead corals as rarefaction curves rapidly reached a plateau (Appendix S2). Coral obligate species were always absent from completely dead corals. On the other hand, our sampling effort was not sufficient to adequately describe non-obligate species communities for any of the coral groups sampled (Appendix S2).

A total of 122 decapod species were collected from 82 corals. We found 55 species on 52 non-eaten corals, 103 species on 22 partially eaten corals, and 62 species on 8 completely dead corals. Interestingly, two crab species, *Nucia rosea* and *Liomera striolata*, and the shrimp *Neostylodactylus* cf. *littoralis* (see photographs in Appendix S3), found on partially dead corals, were previously unknown to French Polynesia.

Numbers of coral obligate species increased with increasing live coral. Coral obligate species represented nearly a third of the decapods collected in non-eaten corals (16 out of 55 species), including nine species of the crab genus *Trapezia* (i.e. *T. areolata* and *T. serenei*, photographs in Appendix S3). However, coral obligates represented only 17% (15 out of 103 species) of the total diversity found in partially eaten corals and none were found on completely dead corals.

**Figure 3:**
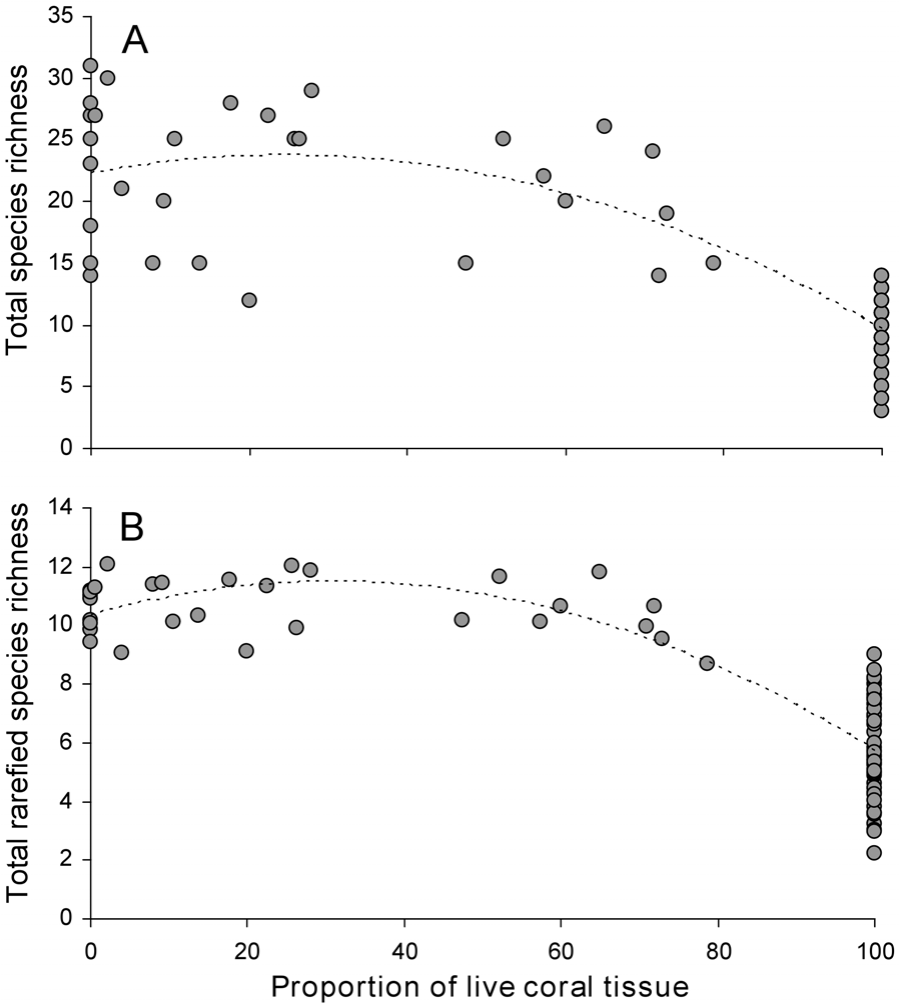
Effect of the loss of live coral tissue on overall decapod species richness. (A) Total decapod species richness: polynomial regression, *y* = −0.0025*x* ^2^+0.1214*x*+22.258; *R^2^* = 0.72, N = 82, *p*<0.001. (B) Rarefied total decapod species richness: polynomial regression, *y* = −0.0012*x* ^2^+0.0746*x*+10.352; *R^2^* = 0.67, N = 82, *p*<0.001.

**Figure 4:**
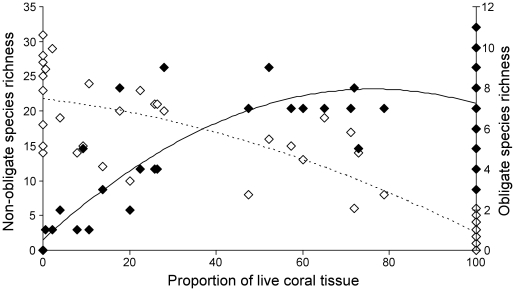
Response of coral obligate and non-obligate decapod species to the loss of live coral tissue. White diamonds and discontinuous line = non-obligate decapod species richness *y* = −0.0012*x* ^2^−0.0737*x*+21.739; *R^2^* = 0.85, N = 82, *p*<0.001. Black diamonds and continuous line = coral obligate decapod species richness: *y* = −0.0013*x* ^2^+0.1951*x*+0.5189 ; *R^2^* = 0.69, N = 82, *p*<0.001.

Second degree polynomial models best fit both the relationship between total decapod species richness and proportion of live tissue (Fig. 3A) and the relationship between total rarefied decapod species richness and proportion of live tissue (Fig. 3B). The negative curves indicate that decapod species richness and rarefied species richness increase with increasingly dead coral tissue. A positive second degree polynomial model best fit the relationship between the proportion of live tissue and coral obligate decapod species richness (solid line in Fig. 4), while a negative second degree polynomial model best illustrates the relationship between the proportion of live tissue and non-obligate species richness (dashed line in Fig. 4). Whilst coral obligate decapod species richness increases with increasingly live coral tissue, non-obligate decapod species richness shows the opposite pattern, increasing with increasing coral mortality.

**Figure 5:**
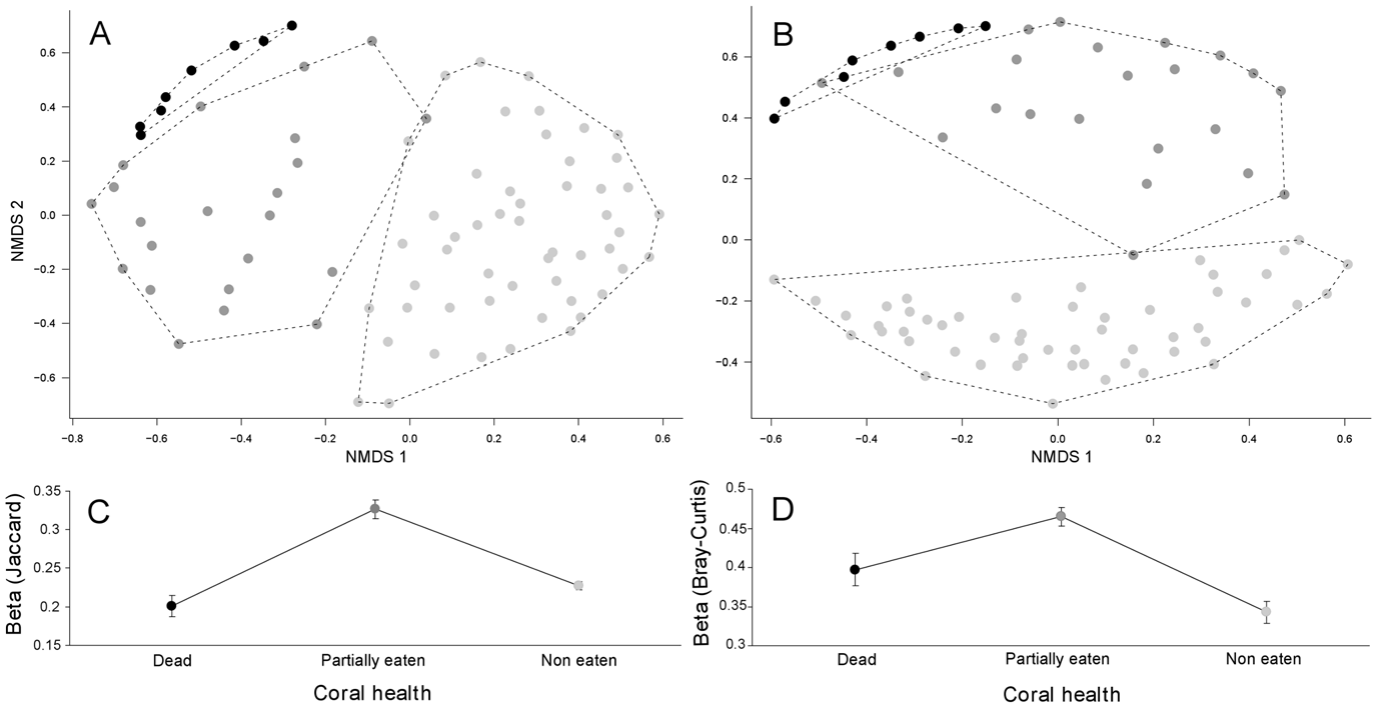
Ordination plots representing the composition of decapod communities with the loss of coral tissue. Non-metric multidimensional scaling plots were computed using Jaccard, an incidence based metric (A) and Bray-Curtis, an abundance based metric (B). Light grey, dark grey and black dots represent non-eaten, partially eaten and dead *Pocillopora* respectively. Mean ± SE beta diversity (average distance to group centroid) is plotted below the corresponding ordination plot (C and D).

NMDS plots show that objects from the three different groups of coral mortality cluster in distinct locations in 2D space indicating compositional differences (Fig. 5A,B). Decapod species composition based on species incidence differed significantly between non-eaten corals and partially eaten corals (PERMANOVA: Jaccard: *F*_1,72_ = 20.11, *p* = 0.001; Fig. 5A), as well as between partially eaten and dead corals (PERMANOVA: Jaccard: *F*_1,28_ = 3.05, *p* = 0.001, Fig. 5A) and between non-eaten and dead corals (PERMANOVA: Jaccard: *F*_1,58_ = 21.64, *p* = 0.001, Fig. 5A). Decapod species composition based on relative abundance showed a similar pattern, differing significantly between non-eaten corals and partially eaten corals (PERMANOVA: Bray-Curtis: *F*_1,72_ = 35.9, *p* = 0.001; Fig. 5B), as well as between partially eaten and dead corals (PERMANOVA: Bray-Curtis: *F*_1,28_ = 3.4, *p* = 0.001; Fig. 5B) and between non-eaten and dead corals (PERMANOVA: Bray-Curtis: *F*_1,58_ = 35.76, *p* = 0.002, Fig. 5B).

Average beta diversity value was significantly higher for partially eaten corals compared to non-eaten and dead corals (PERMADISP: Jaccard: *F*_1,72_ = 41.12; *p* = 0.001 and *F*_1,28_ = 12.65, *p* = 0.001 respectively, Fig. 5C; Bray-Curtis: *F*_1,72_ = 31.42; *p* = 0.001 and *F*_1,28_ = 7.88, *p* = 0.008 respectively, Fig. 5D). There are no significant differences in beta diversity between non-eaten and dead corals either based on species incidence (PERMADISP: Jaccard: *F*_1,58_ = 2.09; *p* = 0.145; Fig. 5C) or based on species relative abundance (PERMADISP: Bray-Curtis: *F*_1,58_ = 2.39; *p* = 0.12; Fig. 5D).

## Discussion

The world's coral reefs are predicted to decline by 40–60% over the next 50 years [65] due to a wide range of perturbations. Shifts from coral dominated reefs to alternative states dominated by algae [66] will affect the diversity and structure of natural communities and thus comprehensive information on ecosystem functioning, with an emphasis on the structure of diversity, is required in relation to habitat change. The effects of loss of coral cover on fish species richness, abundance and composition has been investigated [10]–[12], yet little work has been done on invertebrates species [13], [27]–[30]. This study uses a 10 years dataset to confirm that an outbreak of *Acanthaster planci* is the principal perturbation to corals in Moorea, French Polynesia. Our study also reveals that such a perturbation increases decapod species richness but to the detriment of community composition, with potential consequences for coral functioning and the ability of corals to withstand future perturbations.

The ten year time series analyzed in the present study covers two bleaching events, in 2002 and 2003 [31], which did not cause significant mortality of *Pocillopora* (Fig. 1) in agreement with Adjeroud et al [31]. On the other hand, there was a dramatic reduction in live coral cover of *Pocillopora* spp. (Fig. 1) between 2006 and 2009 corresponding to the outbreak of *A. planci* in Moorea [31], [67]. In 2008–2009, partially degraded corals all had distinct feeding scars (based on high resolution photographs) demonstrating that sea stars played a preponderant role in coral decline during this period (see also [67]). The higher survival rate of larger size corals (Fig. 2) indicates that they are less susceptible to predation by *A. planci*. We suggest that the large branching structure of certain corals may simply prove too difficult to access for corallivorous sea stars. Size has previously been described to play a major role in determining patterns of coral mortality following other short-term disturbances. For example, Bak and Meesters [68] suggested that larger sized corals may be more resistant than smaller corals to non climate stressors such as sedimentation or nutrients. On the other hand, small sized corals had higher survival compared to larger colonies during a bleaching event, indicating that juvenile corals may not always be the most vulnerable [69], [70], [71]. We suggest that the size structure of *Pocillopora* populations at the beginning of an outbreak of *A. planci* may greatly condition the magnitude of coral mortality. Prior to the *A. planci* outbreak in Moorea in 2006 the smallest corals represented between 51–77% of all *Pocillopora* spp. and the reef suffered a 48–77% decline in the proportion of live coral. Here we suggest that *Pocillopora* populations comprised of a high proportion of large sized corals prior to an *A. planci* outbreak may suffer only limited decline in cover and density during an outbreak of the corallivorous sea star.

The consequences of this *A. planci* outbreak on coral “health” (live vs. dead tissue) had important implications for decapod assemblages. Firstly, and contrary to expectations, decapod species richness increases with a decrease in live coral tissue, plateauing at ∼40% live coral tissue (Fig. 3). Whilst this result may appear counter-intuitive, it is better understood if species composition is taken into account. It is the species richness of non-obligate coral decapod species that increases with a decrease in live coral tissue (Fig. 4), whilst the species richness of coral obligate species increases with increasingly live coral tissue (Fig. 4). Our results confirm that habitat specialists (coral obligates) are highly dependent on the health of their habitat making them more susceptible to habitat modifications [6], [7]. Furthermore, considering the known and important functional roles played by coral obligate decapod species [20]–[25], our study shows that habitat perturbations, such as an *A. planci* outbreak, would have severe consequences not only for “habitat formation” (corals) but also for “habitat maintenance” (coral obligate decapods).

At the end of the *A. planci* outbreak in 2009 we found high levels of overlap in species composition, particularly abundance, among non-eaten *P. eydouxi* corals (low mean beta diversity values - Fig. 5C,D) and with none/few non-obligate species present. This overlap in species composition may be explained by complex interactions among coral obligate species (i.e. cooperation) [55] and competitive exclusion of non-obligate species that limit variation in the species pool [72]. However, in corals that had been eaten there was a decrease in coral obligate species diversity which may have been caused by either their direct consumption during coral feeding by *A. planci* or, and more likely, as a result of migration (pers. obs.). As the percentage of live coral decreases, living space declines, increasing the strength of inter- and intra-specific interactions leading to competitive exclusions from the colony and possibly forced migration. Nevertheless, the loss of live coral host tissue and colonization of the skeleton by algae on the same partially eaten *Pocillopora* branching structure created a mixed decapod community composed of both coral obligate and non-obligate species and an increase in species diversity. The consumption of live coral tissue by *A. planci* opened up space for non-obligate species to colonize due to either their preferential use of algal habitats or the reduction in interspecific competition with obligate species that are their superior competitors [53], [72]. Non-obligate species are a highly diverse fraction of coral reef diversity (whose function is largely unknown) that benefit from the physical disturbance caused by *A. planci*.

We also found a high level of similarity (low mean beta Jaccard diversity value, Fig. 5C) among decapod communities inhabiting dead *Pocillopora*. Dead corals hosted a pool of species such as *Chlorodiella laevissima*, *Perinia tumida* and *Athanas djiboutensis*. Importantly, the diversity of decapods living on dead coral heads was largely under-sampled as suggested by rarefaction curves (Appendix S2). This indicates that our sampling effort provides an incomplete representation of decapod assemblages occurring on dead corals. An increased sampling effort on dead corals would have likely strengthened the second degree polynomial models best fitting the relationships between total decapod species richness and proportion of live tissue (Fig. 3A), between total rarefied decapod species richness and proportion of live tissue (Fig. 3B) and between the proportion of live tissue and non-obligate species richness (dashed line in Fig. 4). Therefore, our results are likely to be conservative. The additional decapod species revealed from an increased sampling effort may also have separated out the dissimilarities between dead and non-eaten corals (Figs. 5C,D), but may have reduced the dissimilarity between dead and partially-eaten corals. In any case, we predict that further sampling of dead corals would only serve to strengthen our results and conclusions on the effects of an *A. planci* outbreak on decapods species richness and community composition.

Although our study did not investigate variation in decapod assemblages across a range of coral head sizes, the drastic modifications in decapod communities with the loss of coral tissue is sufficient to indicate that coral obligate decapod populations could only subsist on larger corals left with live tissue on the outer slope of Moorea in 2009. The resilience of large sized *Pocillopora* to *A. planci* outbreaks may have two important implications. Firstly, if larval replenishment of coral obligate decapod species to an island depends mainly upon self-recruitment from the local parental population as has been suggested for fish populations [73], [74], [75], reproductive adults inhabiting these large corals will play a major role in the recovery of local coral obligate populations. Secondly, because larvae of coral obligate species such as Trapeziids, only settle on live coral tissue, only the remaining large *Pocillopora* corals provide favorable settlement substrates [76]. As the reef recovers, juveniles will be potentially able to emigrate to adjacent corals [77] and provide them with their multitude of services. Therefore, not only do we suggest that the size structure of *Pocillopora* populations at the beginning of an outbreak of *A. planci* determines the magnitude of coral mortality after an outbreak, but that the initial size structure of *Pocillopora* populations also determines the resilience of coral obligate decapod species as well.

Outbreaks of *A. planci*, the main cause of coral reef degradation in the Indo-Pacific [32], [78], have profound consequences for the diversity and structure of corals as well as natural cryptobenthic communities. Our study suggests that the size structure of *Pocillopora* populations at the time of an *A. planci* outbreak may greatly determine local persistence and the recovery of corals and their associates. We show that whilst species richness does not decline per se, the community composition of associated decapods changes, as does their functional role, potentially indirectly affecting the persistence of their coral host population, the foundation species of the reef ecosystem. Future studies should now determine the scale and rates of demographic connectivity between coral mutualist populations to better understand the resilience of populations as the frequency, intensity and scale of human-induced perturbations increase.

## Supporting Information

Appendix S1 Description and validation of sampling methodology.
(DOCX)

Appendix S2 Completeness of species sampling effort. Rarefaction curves for the number of coral obligate (A) and non-obligate (B) species as a function of the number of sampled non-eaten (light grey line), partially eaten (grey line) and completely dead (black line) *Pocillopora eydouxi* corals; 95% confidence intervals are plotted for each curve (dashed lines).
(EPS)

Appendix S3 Photographs of decapods sampled in *Pocillopora eydouxi* coral from Moorea. A) *Trapezia areolata*, B) *Trapezia serenei*, C) *Cymo quadrilobatus*, D) *Alpheus lottini*, E) *Harpiliopsis depressa*, F) *Fennera chacei*, G) *Nucia rosea*, H) *Neostylodactylus* cf. *littoralis*, and I) *Liomera striolata*.
(TIF)
