# Supplementary material for: Acanthaster planci Outbreak: Decline in Coral Health, Coral Size Structure Modification and Consequences for Obligate Decapod Assemblages
Source: PLoS One. 2012 Apr 17;7(4):e35456. doi: 10.1371/journal.pone.0035456 (PMC3328453; doi:10.1371/journal.pone.0035456)
Supplement: Appendix S1 — Description and validation of sampling methodology. (DOCX) [file pone.0035456.s001.docx]

*Background*: Decapods associated with *Pocillopora eydouxi* shelter in narrow spaces among coral branches as well as within interstices at the base of coral heads. In order to describe decapod communities associated with *Pocillopora* previous studies [1,2] used exhaustive but destructive sampling methods. Clove oil, a natural anesthetic, has been successfully used in the marine environment to manipulate and/or collect live aquatic invertebrates and vertebrates [3,4,5,6]. A recent field experiment testing the side effects of clove oil showed that deleterious effects on coral growth and survival occurred only after repeated applications of highly concentrated solutions (≥14% clove oil) [7]. Therefore, we tested the efficiency of a sampling method involving a single immersion of coral heads in low clove oil concentration (around 0.02‰) and compared the abundance and diversity of decapods associated with *P. eydouxi* with the total abundance and diversity obtained after complete coral destruction.

*Material and methods*: Five dead and five living colonies of *P. eydouxi* of approximately similar size (30 x 30 x 15 cm) and shape were selected at the study site. Each coral head was enclosed in plastic bags, broken from the substrate with a chisel, placed in 50 L containers and immediately brought to the boat. Most coral heads removed were attached to pieces of rubble that were carefully removed. Decapod and fish symbionts were evicted from their shelters by adding 5 ml of clove oil to 20-30 L seawater in which each coral head was submerged. After one minute, coral heads were then vigorously shaken for 30 seconds to collect individuals still holding on to the branches. Each coral head was then visually inspected and all decapods and fishes were collected with a dip net or with a forceps. These specimens represent the abundance and species diversity using the clove oil method. Each coral head was subsequently broken into small pieces with a hammer and all remaining decapods and fishes were collected. The abundance and species diversity of the two methods combined represents the total abundance and diversity. The efficiency of the clove oil sampling technique was tested using a paired *t*-test comparing the mean diversity and abundance using clove oil only with the total diversity and abundance using both clove oil and complete coral destruction. All collected specimens were initially stored in seawater in a cooler for species identification. Many specimens (at least one per species) were photographed alive to document their live color patterns, which in many cases facilitates species identification.

In order to test the long-term effects on coral survival of the above-described sampling protocol, decapods were collected from 52 live (non-eaten) coral heads which were then attached to cinder blocks, placed in the lagoon, and monitored weekly during the following 5 months (25 weeks).

*Results and conclusion*: The clove oil sampling method was very efficient for sampling all decapod species diversity and abundance in *P. eydouxi* without dead tissue (Table 1). No additional decapod and fish individuals were found after breaking live coral heads, except for two specimens of *Trapezia bidentata* (Infraorder Brachyura) found at the coral head base. However, sampling dead coral heads using clove oil was not as efficient for all species (Table 1). The diversity and abundance of caridean shrimps, as well as diversity of anomurans (predominantly Galatheidae, Porcellanidae and Diogenidae) using the clove oil method were representative of all individuals present (Paired *t*-test: *p* > 0.05; Table 1). However, the clove oil method was not proven to efficiently sample either the species diversity or abundance of brachyuran crabs (Paired *t*-test: *p* = 0.008 and 0.02 respectively) as they often hold firmly onto algae. The clove oil method did not efficiently sample the abundance of anomurans either (Paired *t*-test: *p* = 0.02; Table 1).

**Table 1** – Mean (± SD) species diversity and individual abundance of four decapod sub-orders collected on five live and five dead *Pocillopora eydouxi* in Moorea, using clove oil anesthetization alone and both clove oil anesthetization and complete colony breakdown (Total). Results of paired *t*-tests are given where standard deviation differences among variables were different from 0.

| Coral health |  | Sub-order | Clove oil alone (mean ± SD) | Total (mean ± SD) | *t* |
| --- | --- | --- | --- | --- | --- |
| LIVE | ***Richness*** | |  |  |  |
|  |  | Brachyura | 5.2 ± 1.3 | 5.2 ± 1.3 | N/A |
|  |  | Caridea | 5.0 ± 1.2 | 5.0 ± 1.2 | N/A |
|  |  | Anomura | 0.8 ± 0.8 | 0.8 ± 0.8 | N/A |
|  | ***Abundance*** | |  |  |  |
|  |  | Brachyura | 12.2 ± 6.4 | 12.6 ± 6.8 | 1.63 |
|  |  | Caridea | 36.8 ± 17.0 | 36.8 ± 17.0 | N/A |
|  |  | Anomura | 2.0 ± 2.4 | 2.0 ± 2.4 | N/A |
| DEAD | ***Richness*** | |  |  |  |
|  |  | Brachyura | 8.0 ± 1.6 | 12.0 ± 3.3 | **4.78*** |
|  |  | Caridea | 4.8 ± 2.8 | 4.8 ± 2.8 | N/A |
|  |  | Anomura | 5.0 ± 2.2 | 5.6 ± 1.8 | 2.45 |
|  | ***Abundance*** | |  |  |  |
|  |  | Brachyura | 27.2 ± 12.9 | 42.4 ± 21.9 | **3.77*** |
|  |  | Caridea | 12.4 ± 9.8 | 12.6 ± 10.2 | 1.00 |
|  |  | Anomura | 20.4 ± 10.5 | 24.8 ± 11.6 | **3.92*** |

* = *p* < 0.05, significant differences are highlighted in bold.

**Literature cited**

1. Coles SL (1980) Species diversity of decapods associated with living and dead reef coral *Pocillopora meandrina*. Marine Ecology-Progress Series 2: 281-291.

2. Gotelli NJ, Abele LG (1983) Community patterns of coral-associated decapods. Marine Ecology-Progress Series 13: 131-139.

3. Bower JR, Sakurai Y, Yamamoto J, Ishii H (1999) Transport of the ommastrephid squid Todarodes pacificus under cold-water anesthesia. Aquaculture 170: 127-130.

4. Depczynski M, Bellwood DR (2004) Microhabitat utilisation patterns in cryptobenthic coral reef fish communities. Marine Biology 145: 455-463.

5. Robertson DR, Smith-Vaniz WF (2010) Use of clove oil in collecting coral reef fishes for research. Marine Ecology-Progress Series 401: 295-302.

6. Venarsky MP, Wilhelm FM (2006) Use of clove oil to anaesthetize freshwater amphipods. Hydrobiologia 568: 425-432.

7. Boyer SE, White JS, Stier AC, Osenberg CW (2009) Effects of the fish anesthetic, clove oil (eugenol), on coral health and growth. Journal of Experimental Marine Biology and Ecology 369: 53-57.
