# Supplementary figures and images for: Acanthaster planci Outbreak: Decline in Coral Health, Coral Size Structure Modification and Consequences for Obligate Decapod Assemblages
Source: PLoS One. 2012 Apr 17;7(4):e35456. doi: 10.1371/journal.pone.0035456 (PMC3328453; doi:10.1371/journal.pone.0035456)

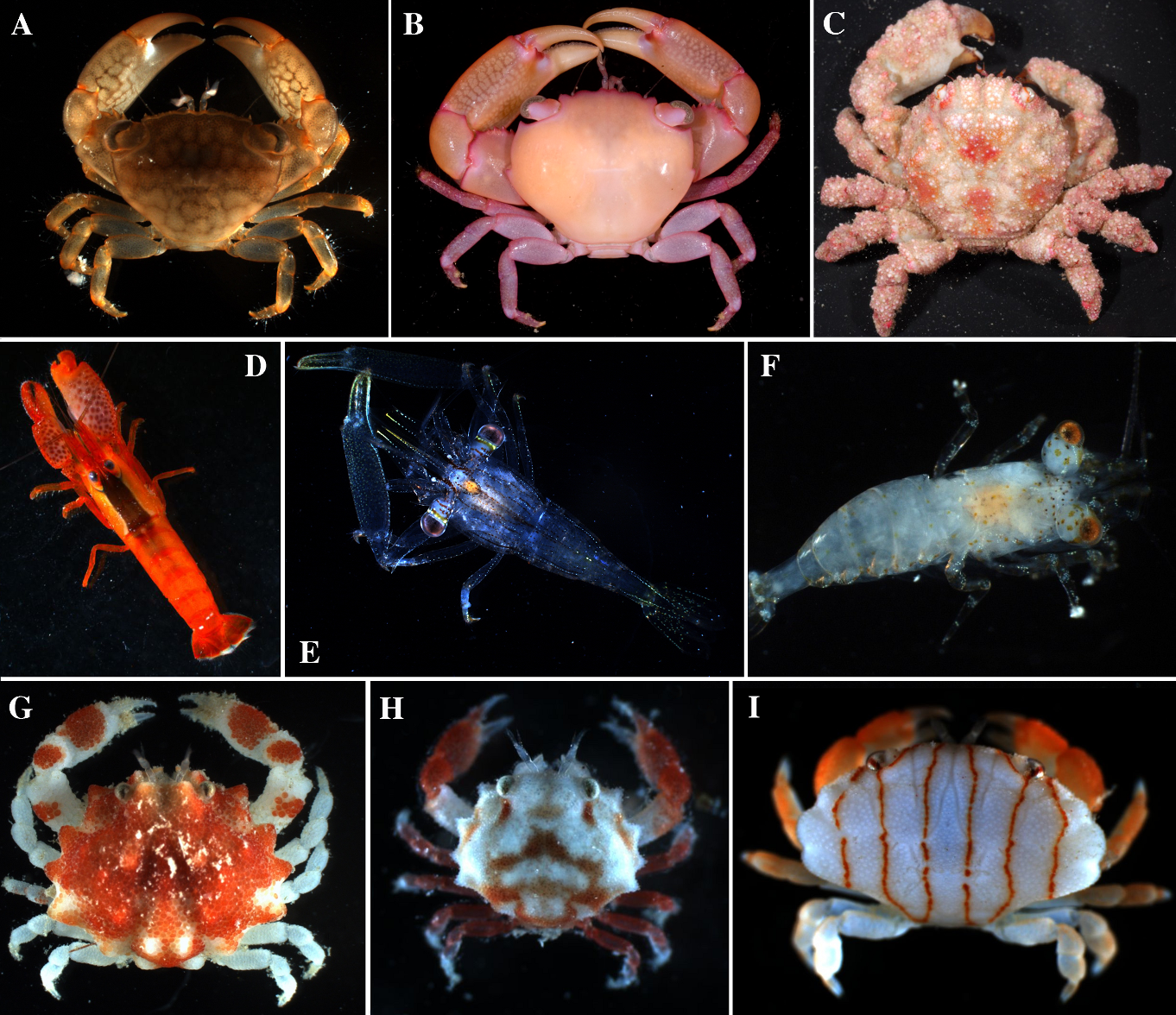

Supplement: Appendix S3 — Photographs of decapods sampled in Pocillopora eydouxi coral from Moorea. A) Trapezia areolata, B) Trapezia serenei, C) Cymo quadrilobatus, D) Alpheus lottini, E) Harpiliopsis depressa, F) Fennera chacei, G) Nucia rosea, H) Neostylodactylus cf. littoralis, and I) Liomera striolata. (TIF) [file pone.0035456.s003.tif]
